# Risk Factors for Respiratory Syncytial Virus–Associated Community Deaths in Zambian Infants

**DOI:** 10.1093/cid/ciab453

**Published:** 2021-09-02

**Authors:** Caitriona Murphy, William B MacLeod, Leah S Forman, Lawrence Mwananyanda, Geoffrey Kwenda, Rachel C Pieciak, Zachariah Mupila, Donald Thea, Chilufya Chikoti, Baron Yankonde, Bernard Ngoma, Charles Chimoga, Christopher J Gill

**Affiliations:** 1 Right to Care Zambia, Lusaka, Zambia; 2 Boston University School of Public Health, Biostatistics and Epidemiology Data Analytics Center, Boston, Massachusetts, USA; 3 Boston University School of Public Health, Department of Global Health, Boston, Massachusetts, USA; 4 University of Zambia, School of Health Sciences, Department of Biomedical Sciences, Lusaka, Zambia

**Keywords:** RSV, Infant Mortality, Zambia, Risk Factors

## Abstract

**Background:**

Respiratory syncytial virus (RSV) is a major cause of infant deaths. Its epidemiology in low- and middle-income countries is poorly understood. Risk factors associated with RSV-associated infant deaths that occur in community settings are incompletely known.

**Methods:**

Community deaths for infants aged 4 days to 6 months were identified during a 3-year postmortem RSV prevalence study at the main city morgue in Lusaka, Zambia, where 80% of deaths are registered. This analysis focuses on the subset of deaths for which an abbreviated verbal autopsy was available and intended to sort deaths into respiratory or nonrespiratory causes by clinical adjudication. Posterior nasopharyngeal swab samples were collected within 48 hours of death and tested for RSV using quantitative reverse-transcription polymerase chain reaction. Associations between potential risk factors were determined as relative risks with 95% confidence intervals (CIs).

**Results:**

We prospectively enrolled 798 community infant deaths with verbal autopsies and RSV laboratory results, of which 62 results were positive. The mean age of the infants was 10 weeks, and 41.4% of them were male. Of all deaths, 44% were attributed to respiratory causes. RSV was detected in 7.8% of the community infants and was significantly associated with respiratory deaths (risk ratio, 4.0 [95% CI, 2.2–7.1]). Compared with older infants, those aged 0–8 weeks had a 2.83 (95% CI, 1.30–6.15) increased risk of dying with RSV. The risk of RSV for the 0–8-week age group increased to 5.24 (1.56–33.14) with adjustment for demographics, parental education, and geography. RSV deaths were increased with domiciliary overcrowding and were concentrated in poor and dense neighborhoods in Lusaka (risk ratio, 2.00 [95% CI, 1.22–3.27]).

**Conclusion:**

RSV is a significant contributor to community respiratory deaths in this population, particularly in the first 3 months of life and in the more poor and dense parts of Lusaka.

In children <5 years of age, respiratory syncytial virus (RSV)–associated acute lower respiratory tract infections are a leading cause of death [1, 2]. The Pneumonia Etiology Research for Child Health (PERCH) study demonstrated that RSV was the largest contributor to acute lower respiratory tract infection cases across several low- and middle-income countries, and had a population-attributable fraction 3 times larger than the next most prevalent pathogen [3]. However, the PERCH study was restricted to hospitalized children and did not measure the fatal impact of RSV in a community setting. Elsewhere, Nair and colleagues [4] have hypothesized that the estimated global burden of RSV-associated deaths may be significantly underestimated, given the lack of accurate data on such deaths that occur in community settings, outside of medical care.

To address this knowledge gap, we conducted the Zambian Pertussis/RSV Infant Mortality Estimation (ZPRIME) study to directly measure the burden of RSV-associated infant deaths in community and facility settings. In an interim analysis, we observed that roughly 70% of RSV deaths occurred in the community [5]. The goal of the current analysis is to better characterize the risk factors associated with these community RSV infant deaths.

## METHODS

### Study Overview

ZPRIME was a prospective postmortem study carried out in Lusaka, Zambia, from August 2017 to August 2020. In Zambia, infants who died in the community are referred to as “brought in dead” (BID). BID infants must first be reported to the police and are then referred to the University Teaching Hospital (UTH) Mortuary to receive a death certificate and a burial permit to allow the body to be interred. Therefore, we concentrated our resources at the UTH morgue, through which approximately 80% of all deaths in Lusaka pass through before burial.

### Eligibility Criteria

**Figure 1.**
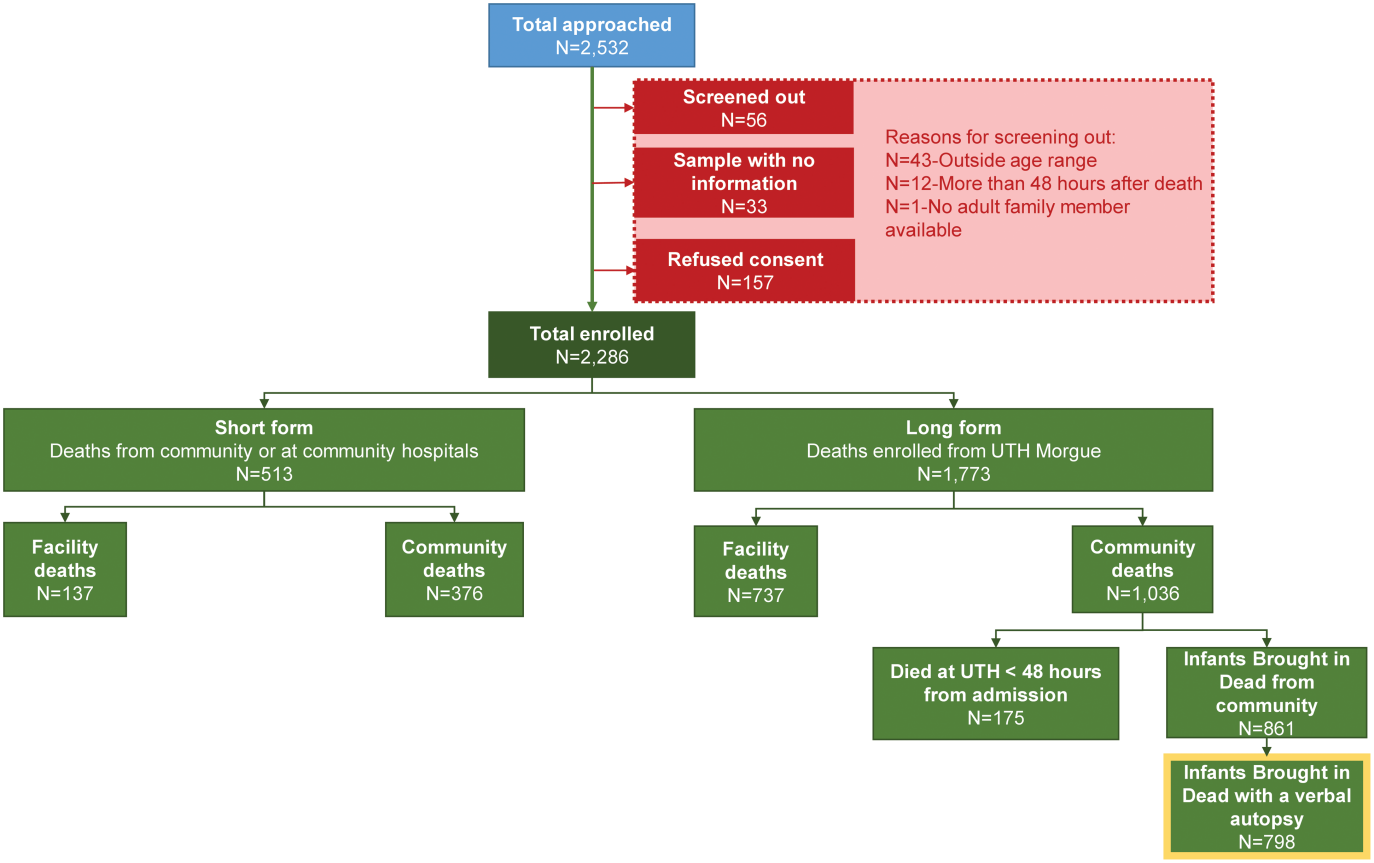
Flowchart depicting entry pathway for final enrollment into the Zambian Pertussis/RSV Infant Mortality Estimation (ZPRIME) study, including sources of noninclusion. Between August 2017 and August 2020, ZPRIME enrolled 2286 infants, aged 4 days to <6 months. We collected “long form” data for 1773 infant deaths; 58.4% of these deaths (1036 of 1773) were community deaths. Among the community deaths, 861 (83%) were in infants “brought in dead” (BID). For the current analysis, we focused on a subset of these deaths (798 of 861 [93%]) for which we had collected long-form data and an abbreviated verbal autopsy. Together, these data were used by study physicians to classify deaths as “respiratory” versus “nonrespiratory.” Abbreviation: UTH, University Teaching Hospital.

Figure 1 describes the pathway for final enrollment into the infant cohort, including sources of noninclusion. Any infant who died at 4 days to <6 months old, for whom we received written informed consent from the infant next of kin, was eligible for inclusion in the ZPRIME study. To reduce the risk of false-negative polymerase chain reaction (PCR) results owing to degradation of viral RNA, we excluded infants who had died >48 hours before enrollment. While most infant deaths are registered at UTH, several other facilities in Lusaka also issue death certificates, albeit at very low volumes.

For deaths occurring at these satellite sites, or during nights, weekends and holidays, we obtained nasopharyngeal swab samples plus very basic demographic infant data (age and sex only), which we termed “short-form data.” For all other deaths at UTH during the week, we concentrated our team efforts on collecting “long-form data.” This contained detailed clinical information on the circumstances surrounding the infant’s death and some demographic and household data. The distinction is that only the long-form data include the detailed clinical and demographic data that are needed to determine associations between RSV PCR status, individual or household characteristics, and respiratory symptom status. Therefore, the current analysis focuses on deaths in BID infants (a subset of community deaths) for which we had collected long-form data and an abbreviated verbal autopsy (see Figure 1).

### Data Collection

When an infant was referred to UTH, morgue attendants notified ZPRIME team members, who then approached the family member to offer grief counseling. Counseling was offered to all families and was not contingent on whether the family consented. On consent, family members filled out an intake form, which provided data on household and demographic features such as parental education.

Copan nylon swabs were used to sample the posterior nasopharynx, because they optimize the yield of detecting respiratory viruses in comparison to the rayon and Dacron swabs [6, 7]. The nasopharyngeal swab samples were stored on ice and transported using universal transport medium to the microbiology laboratory at UTH, where they were aliquoted and stored for later testing. On DNA extraction, RSV was confirmed using a single-plex reverse-transcription PCR assay, with an RSV-positive result defined as a cycle threshold value ≤40. Quality control measures included the use of a positive and negative control, plus, the constitutive human enzyme RNase P, which served to validate the adequacy of sample collection and storage as an internal control for each PCR plate.

### Analysis

Classifications of the cause of death were conducted using data from the verbal autopsies. We used the verbal autopsy tool that was created and validated by the Population Health Medical Research Council. In the ZPRIME study, we used the shortened version of the verbal autopsy tool that was adapted and validated by the Institutes for Health Metrics Evaluation. This tool has been successfully used for infants in Zambia [8]. It includes 60 questions addressing common syndromic causes of death, along with a free-text narrative in which the respondent—invariably a family member—was asked to narrate the circumstances of each infant death. These data sets were subsequently evaluated by 3 study physicians (L. M., D. T., and C. J. G.) to sort them into respiratory or nonrespiratory clinical syndromes. Each physician evaluated each case independently, without knowledge of the PCR results. Discordant results were then discussed at a subsequent reconciliation meeting also blinded to PCR results, leading to a final determination of each case as “respiratory” or “nonrespiratory” deaths; deaths were deemed “uncertain” if the clinicians could not reach a unanimous consensus.

From these data, we conducted descriptive analyses of the cohort, which involved contrasting characteristics of infants who died of respiratory versus nonrespiratory causes, and also those who were RSV positive versus RSV negative. Risk ratios (RRs) and 95% confidence intervals (CIs) were calculated, and differences were considered statistically significant at *P* < .05. Some variables were collapsed into dichotomous categories. Household overcrowding was defined as having >5 members in a household. A primary school has 7 years in Zambia; consequently, educational levels were stratified between ≤7 and >7 years of schooling. Infants were stratified into 3 age groups: 0–8, 9–17, and 18–24 weeks. We also sorted RSV-associated deaths by geographic location within Lusaka, using each infant’s nearest health center as a proxy for their constituency of residence. We note that Kanyama and Chawama are the most densely populated and poorest sections of Lusaka, and areas where access to medical care is particularly limited [9, 10]. Finally, multivariable log- binomial regression was carried out using R software, version 1.4.1103 (R Foundation for Statistical Computing).

### Ethical Statement

The ZPRIME study was approved by the Boston University institutional review boards (reference H-36469) and the University of Zambia Biomedical Research Ethics Committee. Written consent was acquired from the infants’ caretakers.

## RESULTS

From August 2017 to August 2020, we enrolled into the ZPRIME study and performed testing in a total of 2286 deaths, in infants aged 4 days to <6 months. We collected long-form data from 1773 infant deaths, 1036 (58%) of which were community deaths. Among these community deaths with long-form data, 861 were in BID infants. Of these BID infants, we collected an abbreviated verbal autopsy for 798 (93%), who were included in this analysis.

As summarized in Table 1, the median age at death was 10 weeks, with the largest concentration of deaths among younger infants in the 0–8-week age group. Just under half (41%) were male infants. Nearly all infants lived with their biological mother, while roughly three-quarters of fathers lived in the home. There were 57.8% of mothers and 63.4% of fathers who had advanced beyond a primary education. The majority of households (71.9%) did not meet the definition of “large household” (>5 members).

**Table 1.** Demographic and Household Characteristics of the Community Infant Death Cohort

Characteristic	Infants, No. (%)^a^ (n = 798)
Infant characteristics	
Age, wk	
0–8	344 (43.1)
9–17	234 (29.3)
18–24	162 (20.3)
Male sex	330 (41.4)
Maternal characteristics	
Educational level, y	
≤7	312 (39.1)
>7	461 (57.8)
Unemployed	628 (78.7)
Employed	157 (19.7)
Lived with infant	792 (99.2)
Paternal characteristics	
Educational level, y	
≤7	158 (19.8)
>7	506 (63.4)
Unemployed	109 (13.7)
Employed	632 (79.2)
Lived with infant	613 (76.8)
Household size	
>5 members	221 (27.7)
≤5 members	574 (71.9)

^a^Percentages may not add up to 100%, owing to missing data.

After the blinded adjudication process, 284 deaths (36%) were classified as respiratory and 366 as nonrespiratory (46%), and 177 (22%) could not be classified. Table 2 contrasts the distribution of demographic features between respiratory and nonrespiratory deaths. The infants had a mean age of 11 weeks, and, overall, the 2 groups were highly concordant. There was a nonsignificant trend toward young infants (age 0–8 weeks) dying of a respiratory cause, compared with other age strata. Low educational attainment by the father was associated with a 26% increase in the likelihood of respiratory death, and large family size was associated with a 23% increase.

**Table 2.** Risk Ratios for Infant Demographic and Household Features by Respiratory Death Classification

Characteristic	RR Comparing Respiratory and Nonrespiratory Deaths (95% CI)
Infant characteristics	
Age, wk	
0–8	0.89 (.71–1.16)
9–17	1.02 (.81–1.28)
18–24 (reference)	
Male sex	0.90 (.74–1.10)
Maternal characteristics	
Educational level ≤7 y	1.09 (.91–1.31)
Unemployed	0.96 (.78–1.19)
Lived with infant	1.75 (.32–9.59)
Paternal characteristics	
Educational level ≤7 y	1.26 (1.02–1.57)^a^
Unemployed	1.05 (.82–1.35)
Lived with infant	0.87 (.71–1.05)
Nearest health clinic: Kanyama/Chawama	1.00 (.84–1.20)
Large household (>5 members)	1.23 (1.03–1.48)^a^

Abbreviations: CI, confidence interval; RR, risk ratio.

^a^Significant association.

RSV was detected in 62 of the 798 BID infants, for a rate of 78 RSV-associated deaths per 1000 community infant deaths. The study population characteristics stratified by RSV status are presented in Table 3. In contrast to a relatively even distribution of respiratory versus nonrespiratory deaths by age, RSV-associated deaths were heavily concentrated in the 0–8-week age group. More specifically, 72.4% of infants who died with RSV were aged 0–8 weeks, compared with only 44.3% among the RSV-negative infants (RR, 2.8 [95% CI, 1.3–6.2]). The mean age of the infants who died with RSV was 14.2 weeks, with the highest frequency at age 3 weeks (16.1%). The relative risks of RSV death as a function of male sex, lower maternal or paternal educational level, and parental unemployment were not statistically significant. Likewise, household overcrowding did not seem to have a significant effect on RSV status (RR, 0.92 [95% CI, .5–1.6]).

**Table 3.** Risk Ratios for Infant Demographic and Household Features by Respiratory Syncytial Virus Status

Characteristic	RR Comparing RSV and Non-RSV Deaths (95% CI)
Infant characteristics	
Age, wk	
0–8	2.83 (1.30–6.15)^a^
9–17	0.89 (.34–2.34)
18–24 (reference)	
Male sex	0.82 (.48–1.38)
Maternal characteristics	
Educational level ≤7 y	0.80 (.48–1.33)
Unemployed	0.84 (.47–1.49)
Lived with infant	-
Paternal characteristics	
Educational level ≤7 y	0.78 (.38–1.58)
Unemployed	0.58 (.24–1.42)
Lived with infant	1.51 (.79–2.92)
Nearest health clinic: Kanyama/Chawama	2.00 (1.22–3.27)^a^
Large household (>5 members)	0.92 (.53–1.60)

Abbreviations: CI, confidence interval; RR, risk ratio; RSV, respiratory syncytial virus.

^a^Significant association.

Among the adjudicated cases, the association of RSV and in the respiratory deaths was significantly increased (RR, 4.0 [95% CI, 2.2–7.1). Multivariable regression adjusting for age, parental educational levels and employment, large households, and geography (Table 4) increased the risk of RSV in children aged 0–8 weeks to 5.24 (95% CI, 1.56–33.14).

**Table 4.** Multivariable Logistic Regression for Respiratory Syncytial Virus Status in the Infant Deaths Classified as Respiratory Deaths, Controlling for Demographics, Parental Education and Employment and Geography

Characteristic	RR Comparing RSV and Non-RSV Respiratory Deaths (95% CI)
Infant characteristics	
Age, wk	
0–8	5.24 (1.56–33.14)^a^
9–17	2.58 (.69–18.24)
18–24 (reference)	…
Male sex	1.10 (.56–2.31)
Maternal characteristics	
Educational level ≤7 y	0.60 (.24–1.36)
Unemployed	0.88 (.44–2.35)
Paternal characteristics	
Educational level ≤7 y	1.10 (.42–2.71)
Unemployed	0.43 (.07–1.56)
Lived with infant	2.62 (.74–16.28)
Nearest health clinic: Kanyama/Chawama	1.82 (.90–3.74)
Large household (>5 members)	1.38 (.63–3.14)

Abbreviations: CI, confidence interval; RR, risk ratio; RSV, respiratory syncytial virus.

^a^Significant association.

**Figure 2.**
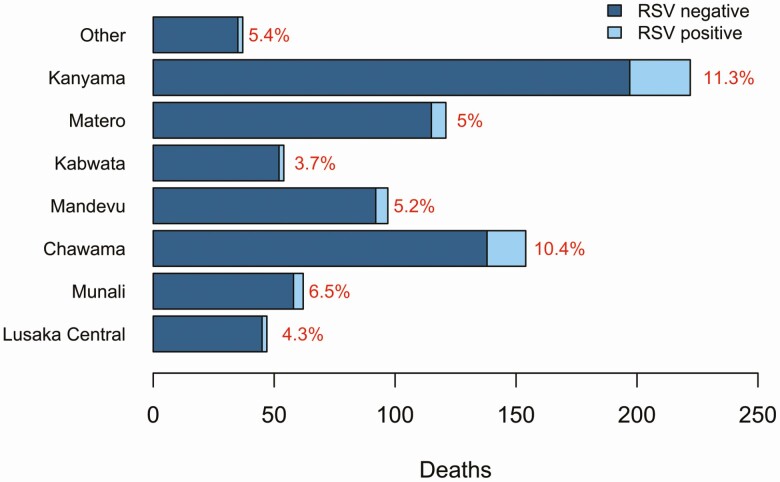
Health facilities were classified into the 7 constituencies in Lusaka District. The frequency of cases by respiratory syncytial virus (RSV) status is presented for each constituency, with the proportion of RSV cases as a percentage at the end of each bar. “Other” represents clinics that were unidentified and were not classified into a constituency.

The distribution of RSV by geographic area in Lusaka is provided in Figure 2. There was a clearly increased concentration of RSV deaths in the Chawama and Kanyama wards. RSV-associated deaths accounted for 11.3% of total deaths in Kanyama and 10.4% in Chawama, constituting a 2-fold increased risk of death with RSV compared with all other constituencies (RR, 2.00 [95% CI, 1.22–3.27).

## Discussion

In this analysis, we found that for every 1000 community infant deaths in Lusaka, approximately 78 were associated with the postmortem presence of nasopharyngeal RSV. Given the strong association between respiratory symptoms and RSV PCR status, it is likely that most of these RSV-associated deaths were caused by RSV. This concentration of RSV-associated infant deaths in a community setting is an important observation that significantly extends our understanding of the fatal burden of RSV in low- and middle-income countries. It is particularly relevant to understanding its implications in terms of why RSV deaths are concentrated in community settings and what our public health responses should be. Regarding the former, we note several contextual factors that may be relevant. A handful of studies carried out in Zambia have identified the prevalence of RSV, but they were all restricted to hospitalized children [11–14]. Thus, the current analysis expands our understanding of the true burden of fatal RSV, highlighting community RSV-associated deaths as the most important source of RSV-associated deaths generally.

Medical management of RSV depends on timely supportive care (supplemental oxygen and suction of respiratory secretions), which may be difficult to deliver in resource-constrained places like Chawama and Kanyama in Lusaka [10, 15, 16]. Both townships have level 1 hospitals (akin to a large clinic with basic inpatient care capacity), but these facilities frequently lack oxygen or suction capacity. Moreover, based on prior work showing that access to care is quite limited and that delays to reaching care are the rule rather than the exception [17], it is predictable that infants frequently do not survive long enough to reach even basic care, let alone necessary interventions such as oxygen and suctioning. Therefore, reducing RSV-associated deaths through greater access and timely care could necessitate not only improving the availability of interventions such as oxygen support but also addressing other barriers to care seeking that are common in these communities, such as acceptability of health care services in terms of attitudes or cultural preferences and a lack of health awareness.

While improving health systems is an essential goal, this is a challenging and long-term strategy, given a lack of resources. Our findings support the short-term imperative for population-wide preventive strategies targeting RSV, such as the use of maternal RSV vaccines or postpartum monoclonal antibodies [18, 19]. Both strategies leverage the overall robust provision of antenatal and routine well-child care, which represent comparative areas of success in Zambia.

Consistent with what is known about the age risks for RSV, our analysis shows a significant risk for RSV-associated deaths during the first 2 months of life. This is compared with estimates in Kenya and South Africa, which had the highest proportions of hospitalization in infants 3–6 months of age [20–23]. Kenya also found a substantial proportion (43%) of RSV hospitalizations in infants >6 months old [24]. Community deaths could be occurring in a younger infants owing to rapid disease progression and fragility, reinforcing the importance of increasing awareness and the need to seek care more quickly. Interestingly, in our analysis, the same age association was not the case when considering all deaths classified as respiratory deaths, highlighting the contribution of RSV to infant deaths in Lusaka.

The highest frequency of deaths occurred at 3 weeks of age. As a result, a vaccine to protect infants early in life would have optimal protection when given before 1 month of age. At present, there are several vaccine approaches in first and second clinical trial phases targeting infants in early life [25]. However, owing to the immaturity of immune systems in newborns, vaccination may not be possible directly after birth [26]. This again supports the practicality of a maternal vaccination strategy. Phase III data for the Novavax maternal RSV vaccine, published in 2020, support the effectiveness of this approach [18].

Infants living in the poorer and densely populated Kanyama and Chawama townships had twice the risk of dying in the community with RSV. Yet these townships are the largest population centers in Lusaka and comprise 35.1% of the <5-year-old age group across all of Lusaka [9]. A World Bank poverty mapping study carried out in 2015 found that Kanyama and Chawama have the second and third highest proportions of people living in poverty in Lusaka [27]. When age, parental education and employment, and household size were controlled for, the risk was reduced to 1.82 (95% CI, .90–3.27). While the association was bordering significance after adjustment, the 11.3% of RSV-associated deaths found in these 2 areas is difficult to ignore. The poverty and dense living conditions likely contribute to RSV-associated deaths.

This study had several limitations. First, our clinical adjudication process relied on the verbal autopsy data provided by nonmedical professionals. As a consequence, there is surely some degree of misclassification of respiratory deaths, which would tend to bias those results to the null and may underestimate the true proportion of infants dying of respiratory causes. By contrast, the analysis by RSV status should be robust because it was based on the results of PCR testing. Second, death in the presence of but not due to RSV likely led to an overestimation of RSV as a cause of death. Third, we used a question from the verbal autopsy about the infant’s nearest health clinic as a proxy for the area of Lusaka City in which they reside. As noted above, this could lead to some minor geographic misclassification.

Fourth, while our data support a causal role of RSV, we cannot exclude the possibility that RSV was a coincidental finding in some cases. However, data from the PERCH study strongly support the assumption that the presence of RSV in the nasopharynx is indicative of clinical disease. In that case-control study, RSV was far more common among children with clinical signs of severe or very severe pneumonia, and comparatively rare in healthy children. We also observed a strong association between respiratory symptoms and RSV status, which is consistent with the PERCH study findings and further evidence that RSV plays a causal role in these deaths.

In conclusion, our analysis confirms earlier observations that RSV is an important cause of community infant deaths and is more frequent in infants <2 months old. It remains to be determined whether this occurs because of more rapid disease progression in these young infants or, alternatively, because of poor or delayed access to supportive oxygen and suction of respiratory secretions in this setting. There was a concentration of RSV-associated deaths in Kanyama and Chawama compounds, and— given the poor access to care and the crowding and poverty—these data further support the need for broad population-level interventions that target this common but preventable cause of infant death.

## Notes

***Financial support.*** This work was supported by the Bill & Melinda Gates Foundation (grant number: OP1163027).

***Supplement sponsorship.*** This supplement is sponsored by the Bill and Melinda Gates Foundation.

***Potential conflicts of interest.*** All authors: No reported conflicts. All authors have submitted the ICMJE Form for Disclosure of Potential Conflicts of Interest. Conflicts that the editors consider relevant to the content of the manuscript have been disclosed.

All authors contributed significantly throughout the study and aided in interpreting results, reading and approving the final version.
